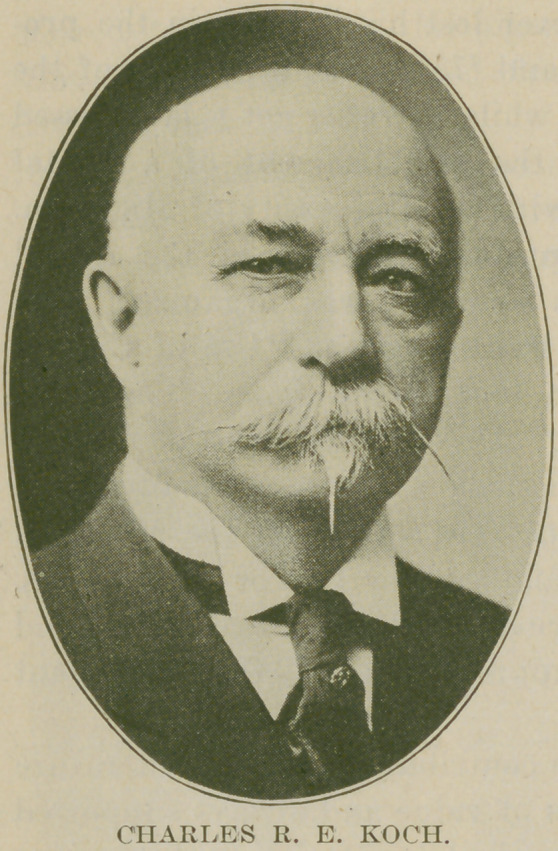# Dr. Charles R. E. Koch

**Published:** 1916-11

**Authors:** 


CHARLES R. E. KOCH.

Dr. Charles R. E. Koch. Doctor Koch was born in Prussia, April 24, 1844, and died in the home of his daughter at Newtonville, Mass., July 21, 1916. Doctor Koch was Secretary of the Northwestern University Dental School of Chicago. He began the study of dentistry in Chicago, with Dr. Kennicott, in 1861, but he enlisted in the Federal army and did not resume his dental studies until 1866. In 1888 he received an honorary degree from the Dental Department of Washington University. He took an early and active interest in dental society work and also in all military affairs, and for the past twenty years he has been a prominent factor in the dental legislation and in college training. He was a man of strong character and determination, and with a spirit of enthusiasm for the cause of dental education he soon became one of the leaders in all kinds of educational progress. In his capacity of teacher and secretary of the Northwestern Dental School he had unusual opportunities for studying the problems of dental education, and because of his long military training he became a great organizer. Because of his close association with a man of great genius, Dr. G. V. Black, the Dean of Northwestern Dental School, he was able to work out in that institution one of the best courses of instruction given in this country. The perfection of the organization is demonstrated by the large attendance and the quality of the graduates sent out by this school.

Dr. Koch was not a scientific worker in the sense that we refer to such men as Black and Miller, but he was a close student of dental science and its art. He was a teacher, a good writer, and a logical and forceful debator in dental meetings. He contributed many papers to our periodical literature, but his greatest effort along this line was his book on the "History of Dental Surgery," published in 1909, which is the largest and most complete history of dentistry ever printed. He also wrote a book on "Illinois at Vicksburg."

Dr. Koch, although greatly occupied with his professional duties found time to do much valuable work for the military interests of his state and the nation. At the time of the "Spanish-American War" he raised a company of soldiers and paid all their expenses for several weeks until the United States Government could assume this responsibility. At the time of his death he had been on a trip to Washington, D. C., in the interest of the Grand Army of the Republic.

Personally, Doctor Koch was highly esteemed by all who knew him. His military relations made a host of friends for him, and his professional relations were of such a wide spread character that he had a large acquaintance, and in all these relations he was greatly esteemed for his devoted interest and characteristic energy in serving in every capacity to which he was called. Those who knew him will testify to his high ideals and personal attachment to his friends and comrade.

Dr. Koch's call seems premature, as apparently he was in normal health, and there seemed to be a longer period of useful ministration open for him. He had so strong a grip on many important professional affairs that his death seems to be a great loss for the dental profession.

It will be difficult to fill his place, in his school work, his military relations, and in his large circle of friends.

## Figures and Tables

**Figure f1:**